# Contingent Kernel Density Estimation

**DOI:** 10.1371/journal.pone.0030549

**Published:** 2012-02-24

**Authors:** Scott Fortmann-Roe, Richard Starfield, Wayne M. Getz

**Affiliations:** 1 Department of Environmental Science, Policy and Management, University of California, Berkeley, California, United States of America; 2 School of Mathematical Sciences, University of KwaZulu-Natal, Durban, South Africa; University of East Piedmont, Italy

## Abstract

Kernel density estimation is a widely used method for estimating a distribution based on a sample of points drawn from that distribution. Generally, in practice some form of error contaminates the sample of observed points. Such error can be the result of imprecise measurements or observation bias. Often this error is negligible and may be disregarded in analysis. In cases where the error is non-negligible, estimation methods should be adjusted to reduce resulting bias. Several modifications of kernel density estimation have been developed to address specific forms of errors. One form of error that has not yet been addressed is the case where observations are nominally placed at the centers of areas from which the points are assumed to have been drawn, where these areas are of varying sizes. In this scenario, the bias arises because the size of the error can vary among points and some subset of points can be known to have smaller error than another subset or the form of the error may change among points. This paper proposes a “contingent kernel density estimation” technique to address this form of error. This new technique adjusts the standard kernel on a point-by-point basis in an adaptive response to changing structure and magnitude of error. In this paper, equations for our contingent kernel technique are derived, the technique is validated using numerical simulations, and an example using the geographic locations of social networking users is worked to demonstrate the utility of the method.

## Introduction

Kernel density estimation is a popular method for using a sample of points to estimate the distribution that generated those points. During application, a probability-related function (i.e. a non-normalized form with normalization applied at an appropriate point in the calculation) known as a kernel is applied to each sampled point and the kernels are summed to obtain an estimate of the original distribution. This estimation technique is employed in diverse fields such as signal processing [1], econometrics [2] and ecology [3].

A subcategory of distribution estimation problems occurs when points are observed with some error. When non-negligible, the errors can lead to biases in the kernel density estimate. Examples of errors include observation bias where points are more likely to be observed in certain regions of the sampling space (independent of the original distribution) or measurement error where, for instance, the observed location of a point has been randomly displaced with known noise statistics from its true location. Methods have been developed for dealing with both these types of errors [4], [5].

One type of error that has not been considered is the case where points within a defined area are nominally placed at a designated location within that area. Examples of these sampling regimes include ecological applications in which observations of species are mapped to predefined geographic entities, such as the center of the region in which the observation took place or to the center of distinct squares of a grid overlaid on that region. Another example is the analysis of types of location data generated by new social websites such as Twitter or Facebook.

This article is motivated by the need to extend the kernel density estimation technique to these forms of sampling. A kernel density estimation method is presented here in which the shape of the kernel for a specific observation is contingent on a known, location-specific “contingency distribution” representing the potential region and probability of where the observation is located. The contingency distribution for a given observation whose precise location is not known, is defined as the set of all areas that could be the location of that observation and their associated likelihoods.

In this new “contingent kernel density estimation” method, the contingent kernel is determined by the convolution of a kernel function and a contingency distribution function. If the sampling errors vary among observations, the form of the contingent kernel will change on an observation-by-observation basis.

This article proceeds in four parts. In the first part we present background material on kernel density techniques and develop the contingent kernel method. In the second, we derive contingent kernels for several common kernel and sampling regime combinations. In the third, we validate the proposed estimation technique using simulated data and demonstrate the utility of the method using location observations gathered from the social-networking site Twitter. In the fourth, we discuss our findings. Our results indicate that contingent kernel density estimation can lead to more accurate density estimates where characterizable error varies across space.

### Background

Assuming a sample of *n* points **X** = {*X*_1_,…,*X_n_*} drawn from an unknown, univariate probability density function 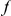. The kernel density estimate 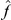 of 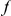 is
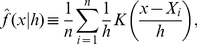
(1)where 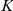 is a user-defined kernel function and 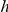 is a user-defined parameter that controls smoothing (often called the bandwidth or the window width). The kernel function may theoretically be of any form such that integration across its domain equals one. In practice, though, kernel functions are typically symmetrical and unimodal around the origin. A commonly used kernel function that satisfies these constraints is the Gaussian kernel, a kernel that is defined as a normal distribution with a mean of zero and a standard deviation of one.

**Figure 1:**
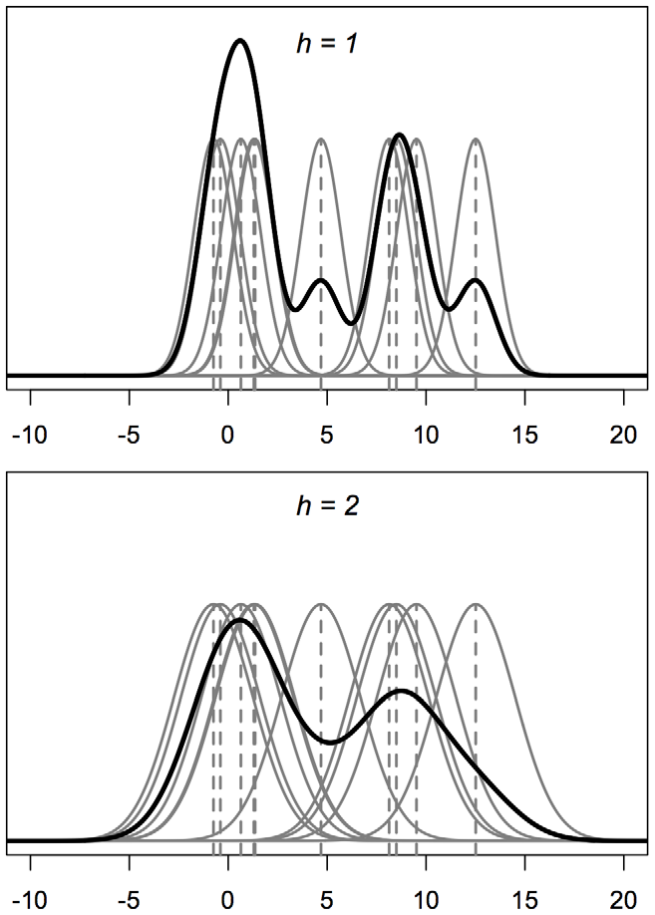
Kernel density estimation illustration. The dark line represents the kernel density estimate while the grey lines are scaled Gaussian kernels for each of ten individual points sampled from the original probability density function. The original probability density function is a mixture function defined as an equal combination of two Gaussian distributions: one with a mean of 0 and standard deviation of 1, the other with a mean of 10 and a standard deviation of 4. The top panel uses a bandwidth of 1 while the bottom panel uses a bandwidth of 2.

Kernel density estimation is a challenging problem, not the least because results are highly sensitive, as illustrated in Figure 1, to the method used to determine the “best” value for the bandwidth parameter 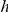 [6], [7]. Beyond this, problems occur when data points are missing, but methods compensating for such error have been developed [5], [8].

Another form of error that has been addressed in the literature is additive measurement error. A number of studies have looked at the case in which the observations can be modeled as the additive effects of the original probability density function and some error probability density function (e.g., [4], [9] and [10]). In this case, the observed distribution of points is the convolution of the original probability density function and the error probability density function. Fourier transformations can be used to develop deconvolution kernels that compensate for the error. The deconvolution kernel density estimator 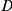 is defined below in Equation 2 where 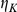 and 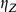 are the Fourier transforms of the kernel 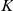 and the error distribution 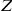 respectively.
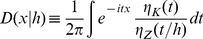
(2)Although conceptually straightforward, the application of deconvolution kernels can sometimes be difficult in practice. For instance, given certain kernel forms, Equation 2 is often intractable to solve analytically. As a result, users may limit kernel choice to aid the development of analytical solutions or apply numerical solution techniques [11].

**Figure 2:**
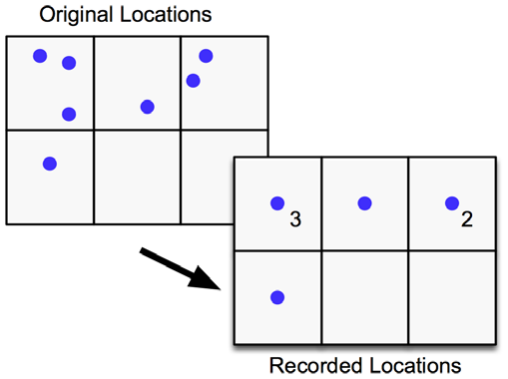
Contamination of observations. An illustration of Transect-Range-Section sampling where the locations of observations within a grid are assigned to the center of enclosing grid squares. Such cases are not suitable for deconvolution techniques to adjust for bias.

Furthermore, the deconvolution methods are not applicable to data gathered in certain common sampling schemes – as when observed points are relocated to the center of the nearest of a set of predefined geographic entities. An example of such sampling schemes occurs when observations are assigned to the center of a Transect-Range-Section grid that has historically been used in the description of locations in species surveys (Figure 2). If a deconvolution kernel were applied to this case, it could result in artificial reductions in density at the centers of the grids, thereby introducing a new form of bias into the estimate (e.g., see [12] Fig. 6.5). Since this form of sampling scheme results in artificial increases in the sharpness of the data, a contingent kernel convolution approach, as described below, that reduces this sharpness is conceptually better than a deconvolution approach which increases sharpness.

In the case of the Transect-Range-Section grid, the geographic entities are all the same size. However, in other sampling regimes this is not the case. For instance, given a statewide survey where observations are assigned to the centers of the nearest county, the size and shape of each county differ. In other cases, the entities themselves may overlap.

Another factor that can lead to differences in the forms of errors is the combination of datasets from multiple surveys. This is especially true for longitudinal surveys for which the duration intersects with the development of new technologies and methods. As an example, take an ecological study that surveys the locations of individual animals of a particular species. If the species is surveyed many times over a period spanning decades, the first surveys may have been carried out using visual identifications, the later surveys carried out using radio collar triangulation, and the most recent surveys completed with GPS sensors. Each technique has its own types of errors. A similar problem arises when combining data from remote sensing satellite systems with different levels of resolution (e.g., MODIS has spectral bands with resolutions ranging from 250–1000 m while Quickbird has a much finer multispectral resolution of 2.4 m).

Unfortunately, many historical ecological datasets are lost or misplaced once their primary investigators move on to other interests. A number of repositories archive these historical studies. The Museum of Vertebrate Zoology at the University of California Berkeley is one such facility containing field notebooks, dating back over one hundred years, that document flora and fauna distributions [13]. The ability to integrate such historical observations with more modern ones under a unified framework is of importance. Such ability would aid in modeling the effects of a changing environment.

To address these issues – to compensate for this form of bias and allow the integration of diverse data sources in a unified analysis framework – we propose in this paper a contingent kernel density estimation methodology developed below. This method extends the application of kernel density estimation to datasets, such as the Twitter data presented later in this document, that previously were problematic to analyze using kernel density estimation.

The contingent kernel is derived by replacing each discrete point location used in standard kernel density estimation with a contingency distribution that represents a local probability density estimate of where the point actually lies. Thus the direct analogue to applying the smoothing kernel to each point as done in standard kernel density estimation, is to apply the kernel to each contingency distribution by convolving the two. Assuming no uncertainty about point locations, this is the case of the Dirac delta function contingency distribution and the contingent kernel method reduces to standard kernel density estimation.

Formally, a contingent kernel 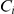 is defined for each point 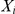 as the convolution of the kernel function, 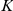, and the probability density function that describes the contingency distribution, Ψ_i_, for that point. Equation 3 defines this operation. Note that the denominator term is a normalizing factor to ensure that the area under the contingent kernel will be one.
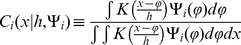
(3)Whereas any standard kernel function can be converted into a contingent kernel through this operation, the contingency distribution is defined by the sampling regime. For instance, in the case of a Transect-Range-Section grid study, the contingency distribution would be a bivariate uniform distribution with the same dimensions as a single grid square.

Once the contingent kernels have been arrived at, the contingent kernel density estimate is calculated in much the same way as the standard kernel density estimate (Equation 4).
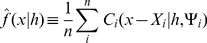
(4)As a practical matter, the form of Ψ*_i_* may often be constant across all points. When this is true, a single form of the contingent kernel function can be derived for all points. This is the case in the Twitter application presented later in this paper. In that application, Ψ is a circular uniform distribution defined by a radius *r* (where *r* may change from point to point). In such cases, a single contingent kernel 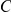 can be derived for the entire dataset, which can then be applied to each point. Here, the primary change from standard kernel density estimation when calculating the density estimate is that the contingent kernel is not based on a one-parameter family of kernels 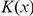, but instead also takes parameters that define the shape of the contingency distribution (*r* in the case of the Twitter application). The rest of the analyses in this paper will focus on cases where the form of Ψ is constant between points.

## Results

### Calculation of Demonstrative Contingent Kernels

**Figure 3:**
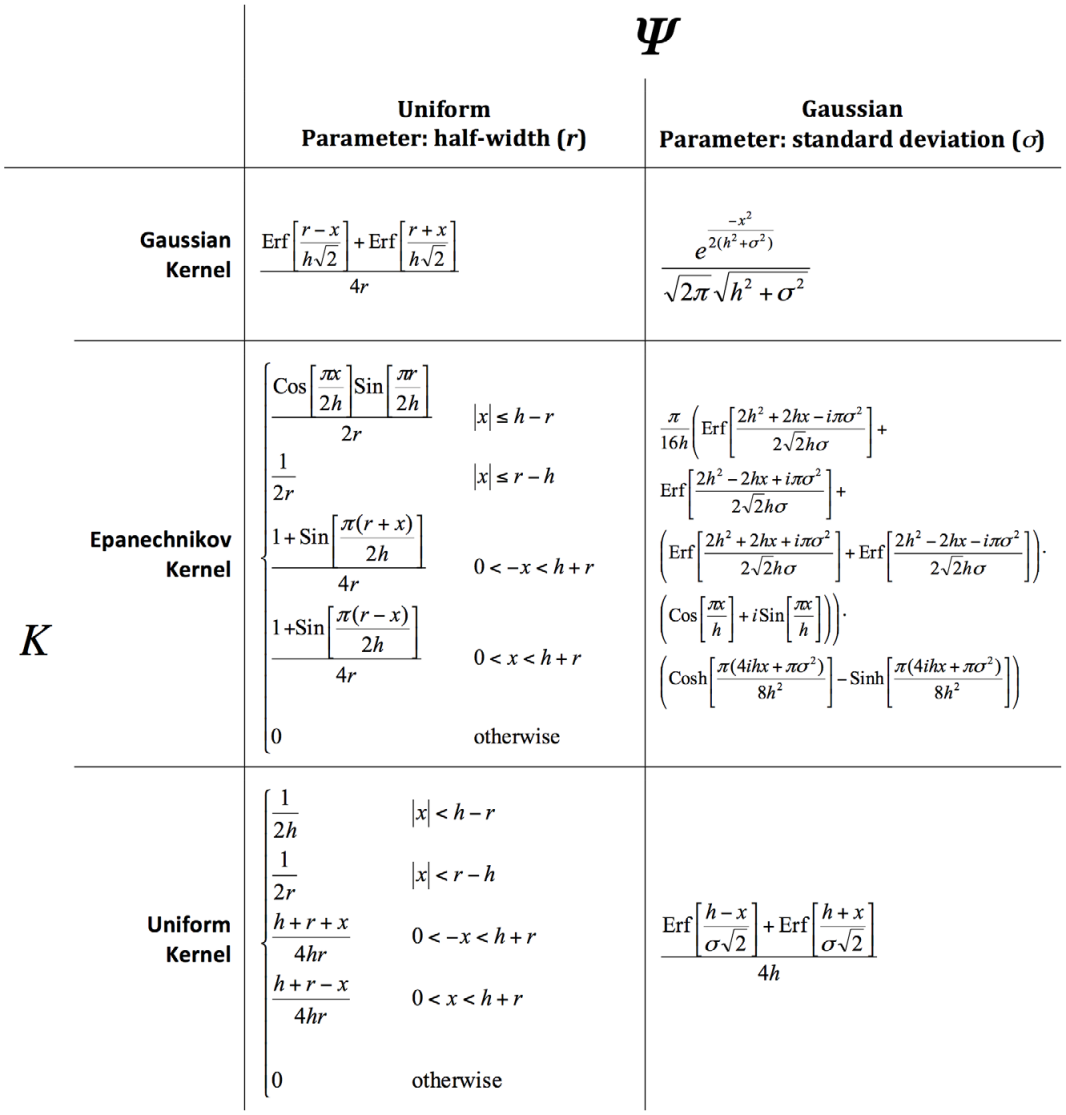
Contingent kernels (*C*) for combinations of univariate standard kernels (*K*) and two forms of contingency distributions (Ψ).

An example calculation of a one-dimensional contingent kernel (as defined using Equation 3) derived for a standard Gaussian kernel and a contingency distribution function (Ψ*_i_*) defined as a uniform distribution with a half-width of *r* (the contingent kernel then depends solely on *r*) is provided by the equation
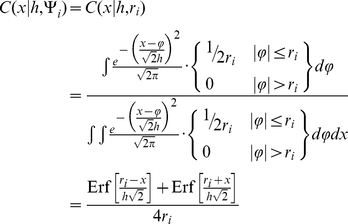
(5)Additionally, contingent kernels for a number of common usage cases were calculated. Combinations between three separate kernels (Gaussian, Epanechnikov, and Uniform) and two types of contingency distributions (Uniform defined by a half width *r* and Gaussian defined by a standard deviation 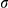) were developed. The resulting contingent kernels are collected in Figure 3.

These contingent kernels directly take the role of the contingent kernel 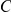 in Equation 4 for calculating the contingent kernel density estimate. In some cases, such as a Gaussian kernel and a Uniform contingency distribution, the contingent kernel is a simple function. In other cases, such as the Epanechnikov kernel and a Uniform contingency distribution, the contingent kernel is a piecewise function where test conditions should be evaluated sequentially until one is determined to be true. In either case, the implementation of these contingent kernels in a mathematical or programming environment is straightforward.

**Figure 4:**
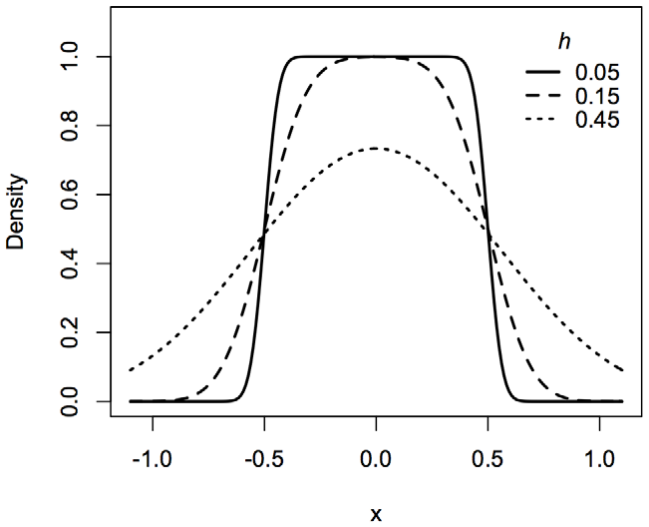
Example contingent kernel. The contingent kernel for a standard Gaussian kernel applied to a uniform half-width of radius 0.5. Contingent kernels for three different bandwidths (*h*) are plotted.

All contingent kernels use the bandwidth as a parameter. Figure 4 illustrates the effect of the bandwidth parameter on the shape of the contingent kernel formed from the standard Gaussian kernel and a Uniform contingency distribution (Equation 5). The parameters defining the contingency distribution are also parameters to the contingent kernels and affect their shape.

It should be noted that not all the contingent kernels are defined for the Uniform contingency distribution when the width of the Uniform distribution is equal to 0 (i.e. *r* = 0). Such a scenario would correspond to the case of no displacement error. Without displacement error, we would wish that the contingent kernel would reduce to the standard kernel that it was derived from. Indeed, this is the case. For example, although Equation 5 is not defined for *r* = 0, the limit of the equation as *r* approaches 0 reduces to the equivalent of what would be the standard Gaussian kernel (Equation 6). Code that implements the contingent kernels should check for such boundary conditions and adjust calculations accordingly.
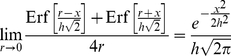
(6)


### Numerical Validation of Accuracy Improvements

As described in the methods section, a numerical simulation was constructed to validate the accuracy of contingent kernel density estimation on synthetic datasets.

**Figure 5:**
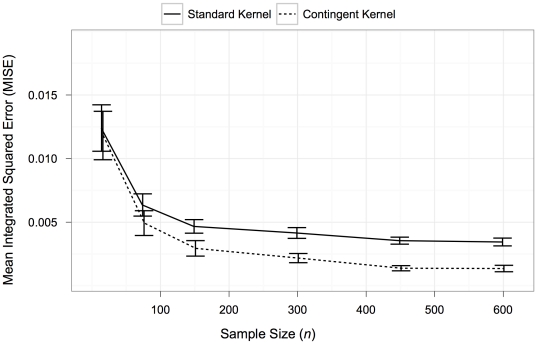
Error for standard and contingent kernels for different sampling sizes from test distribution. Test distribution is a mixture function of three normal distributions. Each normal distribution was sampled with a different frequency. From left to right (2-unit bins, 1-unit bins, and 0.5-unit bins). Mean Integrated Square Error and 95% confidence intervals for the standard and contingent kernels are plotted. 20 Monte Carlo simulations were carried out for each sampling size.

In this experiment, the Mean Integrated Square Error (MISE) of both the standard kernel and contingent kernel estimates fell as the sample size of points drawn from the original distribution increased (Figure 5). Furthermore, although the standard and contingent methods are similar in accuracy for small sample sizes, the contingent kernel error falls faster compared to the standard kernel, as the sample size increases. At large sample sizes, the contingent kernel has approximately one-half the MISE as the standard kernel.

**Figure 6:**
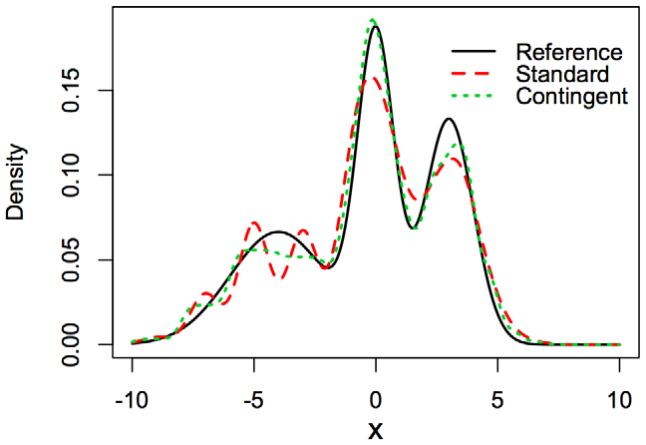
Sample standard and contingent kernel density estimates of test distribution. The distribution is the same as in Figure 5. Sample size is 600 points. The contingent kernel has significantly lower error compared to the standard kernel.

A better qualitative understanding of the methods is obtained by looking at the exact estimates produced by the standard and contingent kernel techniques (Figure 6). The standard kernel method is more susceptible to overestimation of the density at the locations where the points are relocated to, due to the sampling grid filter. The standard method also exhibits higher variability. The contingent kernel method, on the other hand, reduces variability at these locations by smoothing the kernels based on uncertainty. Even as it smooths in these places, the contingent kernel method still does a better job than the standard kernel method at identifying the spike in density at location 0. Since that peak is not associated with high sampling uncertainty, little contingent smoothing occurs at the peak.

### Application: Twitter User Locations

**Figure 7:**
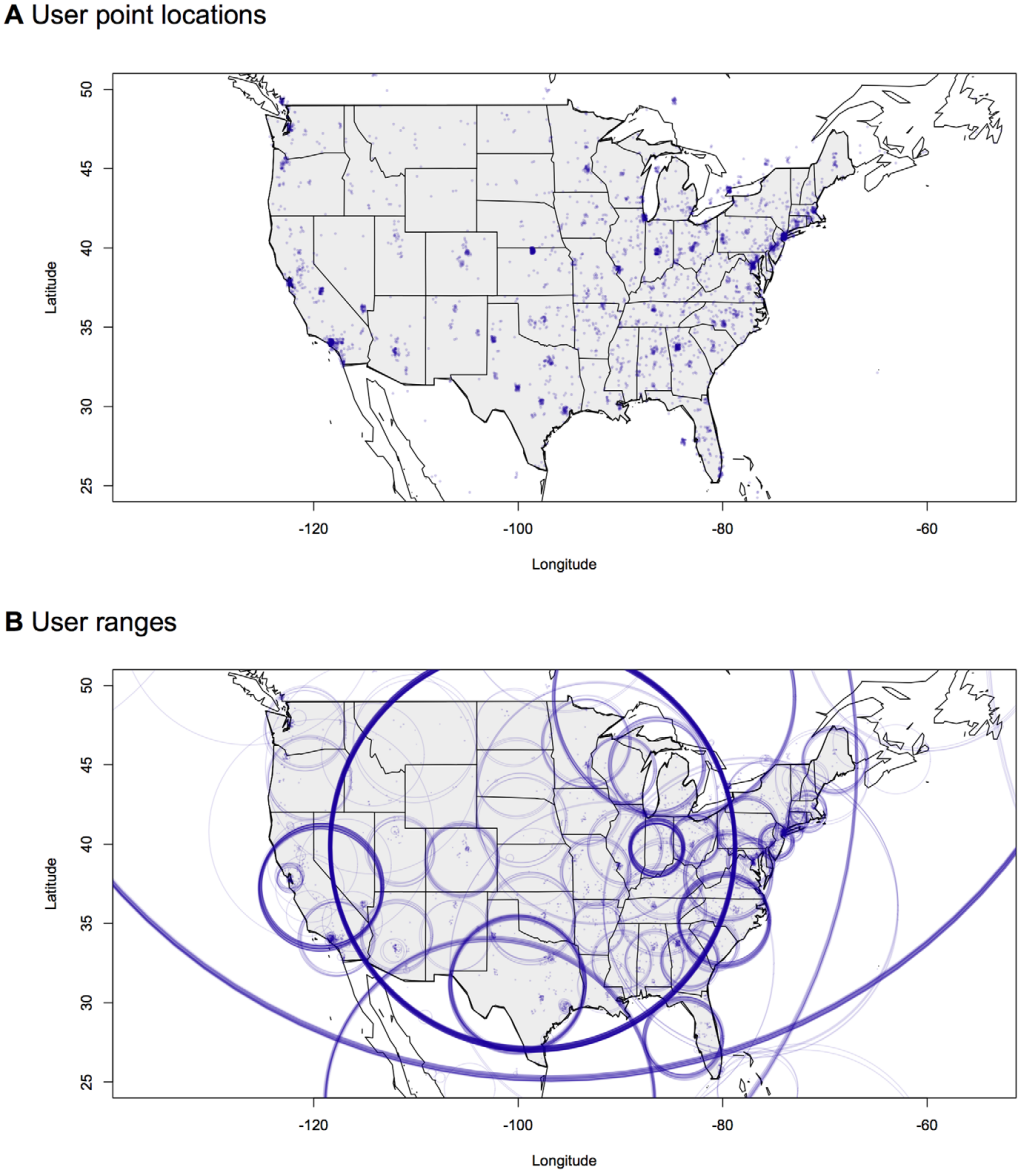
Plots of a subsample of 10,000 Twitter user locations. Panel A is the plot of just the center of the locations. This is what a standard kernel estimate would use to estimate distribution. Panel B is a plot of the discs defined by the center and the radius of uncertainty. This is what the contingent kernels use to estimate distribution. In panel A, note the high concentration of users on the Kansas-Nebraska border. This is due to users entering “USA” as their location and should be paired with high uncertainty. It is converted to the large ring shown in panel B. Points and discs are jittered by up to 2 degrees to improve the readability of the results.

Data from 10,000 Twitter users were analyzed. As discussed in the methods section, location information for each user was represented as a disc with the user having equal probability of being located anywhere in the disc. Figure 7 contains plots of both the center point for each user and the full disc that defines the location of the users. Points and discs are jittered and plotted with slight transparency to allow the estimation of the number of users at dense locations.

Standard kernel density estimation uses the center of the discs. As can be seen in panel A of Figure 7, using this criterion there appears to be a heavy concentration of users directly on the Kansas-Nebraska border. In reality, there is no such heavy concentration of Twitter users in that location, instead the heavy density there is due to users who specified their location as “USA.” The Kansas-Nebraska border is the rough center of the United States and so Twitter users whose location is only identified as the country are assigned to that location. When, the discs are viewed in panel B of Figure 7, the spike on this border disappears as it is transformed to a set of large discs encompassing most of the United States. Similar spikes in densities can be seen in panel A at the center of California or in the center of Texas. When the discs are plotted instead, these spikes disappear and become discs approximately encompassing these states.

**Figure 8:**
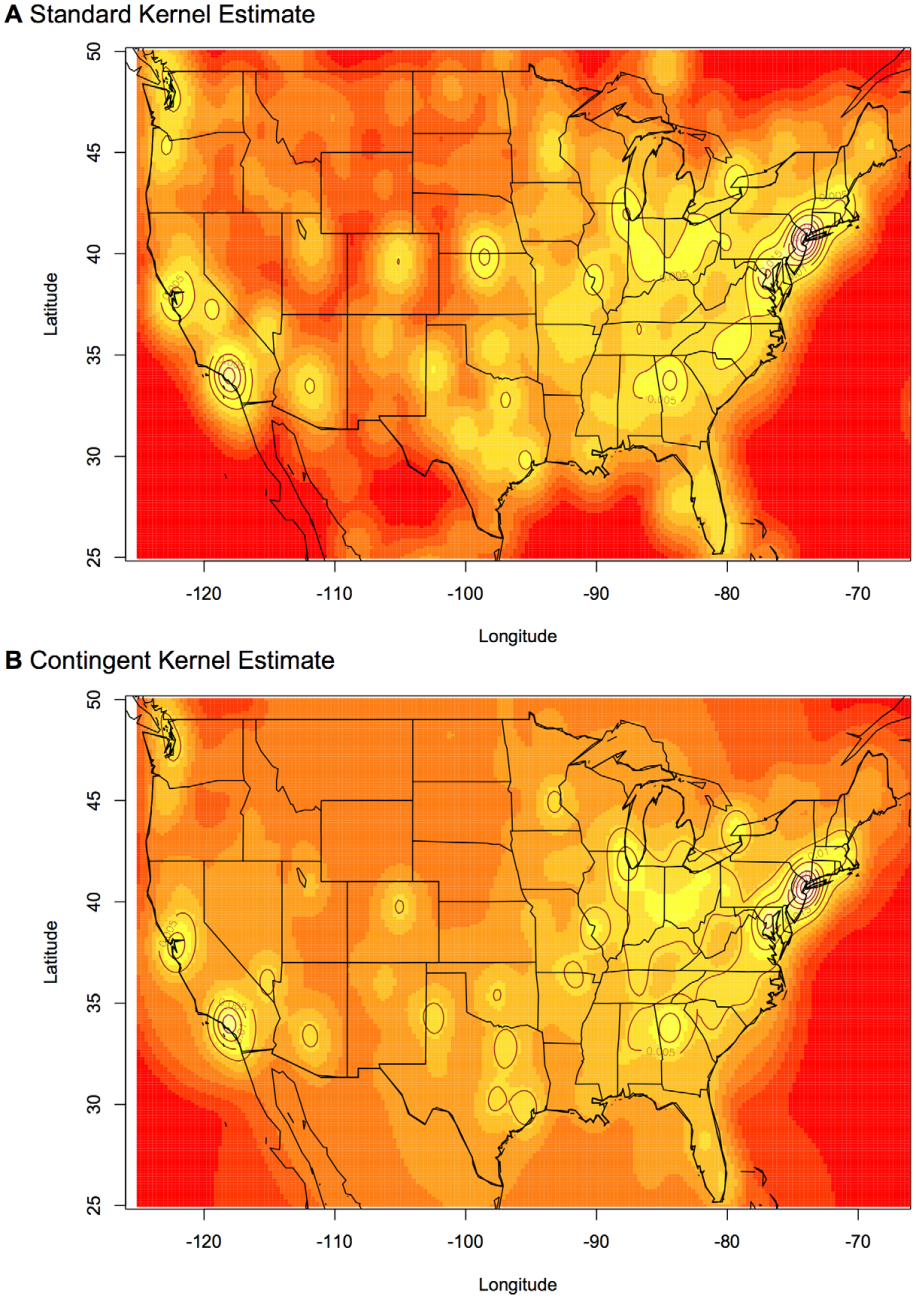
Standard and contingent kernel density estimates of the distribution of 10,000 Twitter users in the United States. Note the high-level of density on the Nebraska-Kansas border found in the standard kernel estimate. In the standard kernel, this level of density is equivalent to the level of density in the San Francisco Bay Area: an area with a known high-level of Twitter use. This is an artifact caused as a result of failing to take the uncertainty of users locations into account. The artifact disappears in the contingent kernel estimate.

Standard and contingent kernel density estimates were carried out for these data using a bivariate Gaussian standard kernel and its contingent kernel equivalent (a circular rotation of Equation 5 normalized so the volume under the kernel is one). The standard kernel density estimate clearly demonstrates strong bias and inaccuracy in that the spike of users who select the United States as their location is represented in the standard kernel density estimate as a large increase in density on the Kansas-Nebraska border (Figure 8). This density spike is approximately equivalent to the spike in density located at the San Francisco Bay Area, a region that is the headquarters of Twitter and that is known for its relative high population of technologically savvy Twitter users. Clearly this is quite unrealistic and is just an artifact of the sampling regime.

In the contingent kernel density estimate, this sampling artifact completely disappears. In addition, population densities and cities with an expected high number of Twitter users such as Denver, Colorado, are better identified in the contingent kernel density estimate than in the standard kernel density estimate.

## Discussion

The contingent kernel density estimation technique has been shown to be effective in compensating for certain forms of errors such as those observed in the Twitter location dataset or those associated with Transect, Range and Section grids. The proposed method allows the integration of diverse data sources, each generated with various levels of measurement confidence and potentially different types and structures of uncertainty. Furthermore, it is more straightforward to analytically derive contingent kernels for standard kernels and elementary contingency distributions than it is to derive some other adjustment methods such as deconvolution kernels. As contingency distributions become more complex (for instance when the contingency region is defined using an arbitrary geographic boundary such as a country border), deriving contingent kernels analytically may be infeasible but numerical approximations can be used to estimate the contingent kernel.

A primary limitation of this method now needs to be addressed: the contingent kernel is dependent upon the contingency distribution Ψ, but this distribution is often not known. In the examples presented here, the contingency distribution was taken as uniform function (the computer simulation validation experiment) and as a uniform disc (the applied Twitter analysis). Given the respective displacement functions, these are the best guesses of the contingency distribution prior to analysis of the data or knowledge of 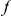. However, if we denote these uniform contingency distributions as 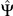 the contingency distributions that appear in Equation 3 are given by
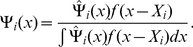
(7)Since 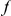 is unknown, Ψ*_i_* cannot be directly calculated. By using 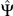 *_i_* in place of Ψ*_i_*, the results are biased. The significance of this bias depends on both 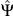 *_i_* and 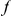. As shown in the computer simulation and in the applied Twitter application, even given the use of 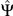 *_i_*, the contingent kernel density estimation can still result in superior density estimates as compared to standard kernel density estimation. This is because in these cases 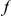 has a relatively small effect on Ψ*_i_* so 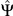 *_i_* and Ψ*_i_* are quite similar. However, as 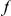 becomes more aggregated relative to 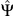 *_i_*, the quality of substituting 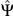 *_i_* for Ψ*_i_* is reduced.

A subsequent goal is the determination of better approximations of Ψ*_i_* from 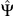 *_i_* and the data. One possible method that this paper has not explored, but which is widely used in problems without closed form solutions, is to carry out an iterative analysis. For instance, we suggest one possible smoothing technique in Equation 8. Here we denote Ψ*_i,0_*(*x*) the first guess of the contingency distribution for a given point *X_i_* (which can be uniform or not) and 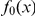 as the resulting estimate of the true 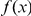. We define the parameter *a* as a real-valued number between 0 and 1 that determines the magnitude of the effect of iteration. When *a* is 0, no iteration is carried out. When *a* is 1, the iteration has maximum effect (and the final form of 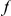 may never converge).

Equation 8 is initially used with *j* = 1 to obtain Ψ*_i,1_* and then use the set of Ψ*_i,1_* to obtain the next estimate 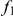 of 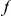. Now repeat using Equation 8 to iteratively obtain a sequence of estimates (Ψ*_i,j_*(*x*), *f_j_*(*x*)), *j* = 1,2,3,… .
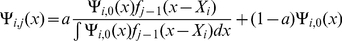
(8)Equation 8 can be decomposed into roughly three conceptual steps. The first step (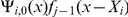) takes the estimate of a contingency distribution and scales it by the estimate of 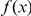. The second step is to normalize the resulting function so it is a distribution with an area of one. Conceptually we can think of this operation as follows: imagine that we had a country with one major city in it. People may either identify themselves as coming from the city or coming from the country. Everyone outside of the city will list the country as their location, while people inside the city will split in listing their locations. Some list the city and some list the country. If we do not take this into account, our estimates will be biased to overpredict the number of individuals outside of the city and underpredict the number within the city. The scaling of the contingency estimates in Equation 8 is one way to approach this issue. The third and final step in Equation 8 is to combine our scaled contingency distribution with the original contingency distribution using a ratio of *a*. As demonstrated numerically in Figure S1, selecting *a*>0 allows the iterative process to converge in some cases while *a* = 0 does not converge.

It should be noted that Equation 8 is presented solely as an illustrative method, its consequences have not been thoroughly explored. One key aspect of this iterative method is the choice of *a*. In practice *a* might be found using some form of cross validation technique. Whether or not this sequence converges to the true contingency and population distributions will generally depend both on the starting distribution Ψ*_i,0_*(*x*) and the true contingency and population distributions.

In general, it can be assumed that any reported measurement was made with some error. Sometimes these errors are purely additive such as when observations were displaced using a normal error distribution from their original location. Other times, errors are a result of a procedure where observations have been aligned to a grid such as in the computer validation example developed in this paper. In other cases, errors may be due to selected effort or observation bias leading points to be detected with different frequency in different regions. Whatever the form, whether or not these errors have a significant result on any analysis must be evaluated on a case-by-case basis. If it is determined that the errors could significantly impact the analysis, the choice of analytical tools must be adjusted to account for this impact. This paper develops one such tool: a new method for adjusting for errors that are found in certain types of sampling regimes. Studies that use such sampling regimes can make use of this technique to improve the accuracy of their analyses.

## Methods

The methods consists of three sections: the calculation of example contingent kernels, numerical validation of accuracy improvements, and an application to the location of Twitter users.

### Calculation of the Demonstrative Contingent Kernels

Mathematical analyses were carried out both by hand and with the use of computer software. Specifically, the software program *Mathematica* version 8.0.1 was used as an aid to the symbolic analyses and the calculation of contingent kernels for common usage cases [14].

### Numerical Validation of Accuracy Improvements

Computer simulations were carried out to numerically validate the improvement of the contingent kernel method as compared to standard kernel methods. The programming environment R version 2.13.0 was used to develop the analyses [15].

The validation process required four components:

1. the original probability density function 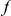 that the two methods attempted to estimate,
2. the sampling protocol which introduced errors into the observed locations of points,
3. the implementation of the standard and contingent kernel density estimation methods including the selection of a kernel and bandwidth, and
4. an error criterion to compare the accuracy of the original probability density function to the estimates 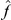.

With respect to point (1), an equal mixture function of three Gaussian kernels *N*(*μ*,*σ*) was used with the following means *μ*, and standard deviations *σ*: *N*(−4,2), *N*(3,1) and *N*(0,0.75) (see Reference curve in Figure 6). Other studies assessing the accuracy of kernel density estimation have looked at mixture functions of Gaussians [16]–[18] and experimentation with several other forms of functions did not change the results.

With respect to point (2), a filter was applied to the sampled observations in order to recreate the type of sampling behavior associated with Transect-Range-Grid sampling and other types of sampling with similar effects. A grid of equally spaced locations was overlaid on the range of the mixture function and the observations drawn from the mixture function were relocated to the nearest grid location. In order to simulate heterogeneous sampling behavior (e.g., if some data are assigned to the county level, while other data are assigned to the state level), three different resolutions of grids were used: the first with a spacing of 0.5, the second with a spacing of 1, and the third with a spacing of 2.

With respect to point (3), the kernel density estimation methods were implemented in the R programming environment. To apply the methods, both the kernel form and the bandwidth value had to be specified. The Gaussian kernel, as it is commonly used, was selected for the analyses. The selection of the kernel bandwidth is a critical issue (see Figure 1) and since the task is a somewhat subjective choice it has received considerable attention in the literature (e.g., see [19] and [20]). In our analysis, for consistency, we selected the bandwidths that minimized in each case the error between the original distribution 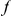 (which we know by construction of our artificial dataset) and its estimate 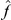, thereby resulting in different bandwidths for the different cases.

Finally, with respect to point (4), errors were assessed quantitatively and qualitatively. Several methods for quantifying errors have been proposed [21], [22]. A common method is to take the squared deviation between the original and estimated distributions. To assess deviations, errors were calculated using the Integrated Squared Error (ISE) (Equation 9 below) between the original and estimated probability density functions for a given dataset **X**.
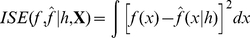
(9)Sensitivity tests compared the error to the sampling size for sample sizes of different datasets **X** ranging from 15 to 600 points. Mean Integrated Squared Error (MISE) over all datasets **X** (i.e. 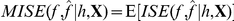) and the 95% confidence interval for this estimate were calculated for each sample size with 20 replicates. Plots comparing the resulting estimates are also shown to provide a more qualitative understanding of the form of these errors.

### Application: Twitter User Locations

To demonstrate the utility of our contingent kernel density estimation procedure, we applied it to location data gathered from the social networking site, Twitter. Location data were collected for 10,000 Twitter users using Twitter's publically accessible *Streaming API*. Only data that users chose to be publically accessible to third party companies and researchers were collected and identifying information was deleted prior to analysis.

Twitter provides two forms of location information from those users who choose to make it publically accessible. The first is precise geolocation data obtained using a GPS device. The majority of Twitter users either do not have such devices or do not choose to make those data public. The more common form of location data is a free form, user-enterable text string describing a user's location.

This location string is definable by the users with little to no restrictions and thus there are high variations in the strings' precisions. For instance, take three hypothetical users living in San Francisco; one user might enter “San Francisco, California,” another “California,” and the third simply “USA.”

Regardless of users' choice of precision, the first challenge in processing the data was to convert the strings to geolocation coordinates that could be mapped and compared. Such a task is non-trivial and either requires extensive manual labor or a large database of place names along with flexible text parsing software (e.g., the software needs to be able to determine that “SanFran, California” and “USA, San Francisco” refer to the same place).

To convert Twitter location strings to geolocated entities, *Yahoo PlaceFinder*, a free service provided by Yahoo, was used. Researchers can send *Yahoo PlaceFinder* free-form location strings, such as those strings entered by Twitter users. The service then processes the information and returns geolocation data for that string. The geolocation information is returned in the form of a disc defined by its center (as a latitude and longitude) and a radius (in meters). For example, if the user entered “San Francisco,” the center of the city and the approximate radius of the city would be returned. If, on the other hand, the user entered “USA,” the center of the country would be returned along with a much larger radius representing and average radius of the country. It is assumed that there is equal probability of the user being anywhere within that resulting disc. A more refined approach could use actual normalized density maps (i.e. converted to the probability of locating individuals in named region at a particular point *x* in that region), when available for the different localities, as the underlying kernels. For purposes of demonstration of our contingent estimation method, though, we simply used a Gaussian kernel basis for the estimation process. We implemented in R both bivariate standard and contingent kernel density estimation techniques for the sample of 10,000 Twitter users. In comparing the results, as discussed below, we identified some critical differences.

Several critiques can be made of using one of the standard family rather than special sets of kernels that conform to regional boundary constraints in our contingent kernel density estimation, as would be the case if kernels were normalized population density maps used for each named region. Even if density maps were not available, it would still be better to use a compact kernel that coincides with the boundaries of the named region in place of a disc that is maybe a very poor approximation to this boundary. Currently the boundaries for certain classes of regions, such as municipalities, are difficult to obtain and identify from the user strings. Also, the use of a set of special but irregular kernels would require a numeric approach, instead of an analytical one, which would greatly slow down computation of a contingent kernel estimate using Equation 4.

Another potential critique is that instead of assuming an equal probability of a user within a region, census or other demographic data could be used to create more accurate predictions of the distribution of users within these regions. This modified approach has two primary potential weaknesses. One is that the distribution of Twitter users could be fundamentally different from the distribution obtained by a census. Factors that result in an individual using Twitter could potentially also result in them having a different geographic distribution than general surveys report for the population at large. Secondly, it can be hypothesized that the level of specificity with which a user enters their location depends on that location itself. For instance, take two hypothetical users living in California. One lives in San Francisco while the other lives in a small town in the Central Valley. A priori, one could hypothesize that the user living in San Francisco would be more likely to specify their city (because they know other Twitter users will have heard of it and so it would then be meaningful to them) while the user living in the small town would be less likely to specify their town (because they know it would likely not have meaning to other Twitter users). If this hypothesis were true, the usage of demographic data could result in bias towards large and popular cities and regions to a greater extent then the assumption of a uniform distribution throughout a region.

## Supporting Information

Figure S1 **Numerical demonstration of convergence of** **equation (9)** **.** A simple sample set 1-D of points was created with the following locations: −1, 1, 1, 2 and 3. A uniform contingency distribution was assumed for each point with radiuses of, respectively, 1, 2, 0.3, 4 and 2.5. The analytical method may only be used for the first estimate of *f* (i.e. *f_0_(x)*) as after that the contingent kernel estimates take on forms not tractable for analytical solutions. The initial contingent kernel density estimate is shown. Iterations were then carried out using different values of *a*. Values of 0.10, 0.50, and 0.75 are shown. Up to 10 iterations were carried out. Plots of iteration estimates of *f* are shown. Iterations are not shown if no significant visible changes were made between iterations. An ellipses sign marks these gaps. As is demonstrated, small values of *a* led to rapid convergence while values closer to 1 led to slower convergence. During iterations regions of high density increase in density while regions of low density generally decrease.
(DOC)
