# Supplementary figures and images for: Contingent Kernel Density Estimation
Source: PLoS One. 2012 Feb 24;7(2):e30549. doi: 10.1371/journal.pone.0030549 (PMC3286465; doi:10.1371/journal.pone.0030549)

**Figure S1**

**Initial Contingent Kernel Density Estimate**


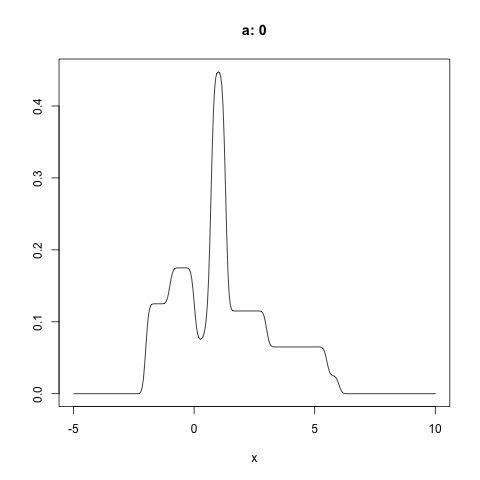


**a = 0.1**


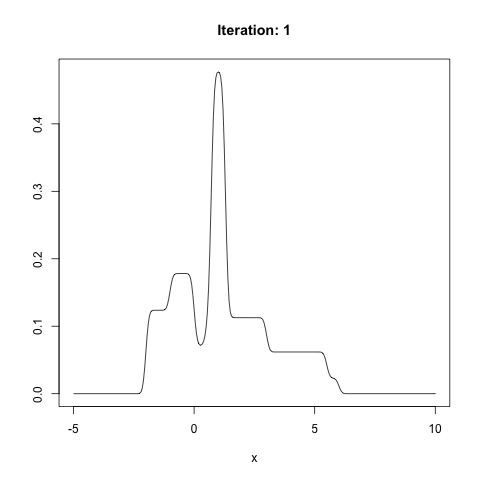

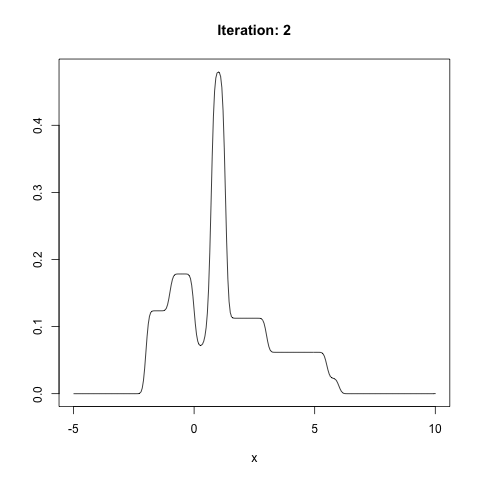


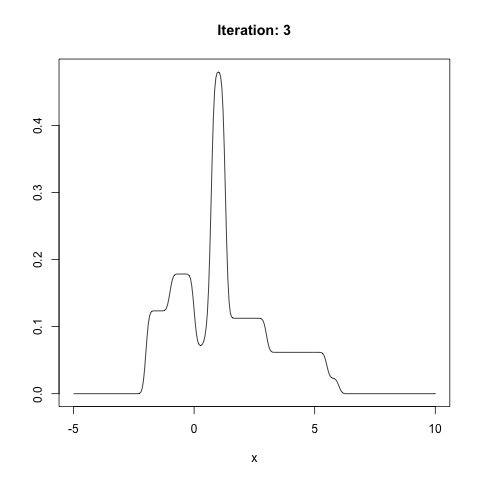
…
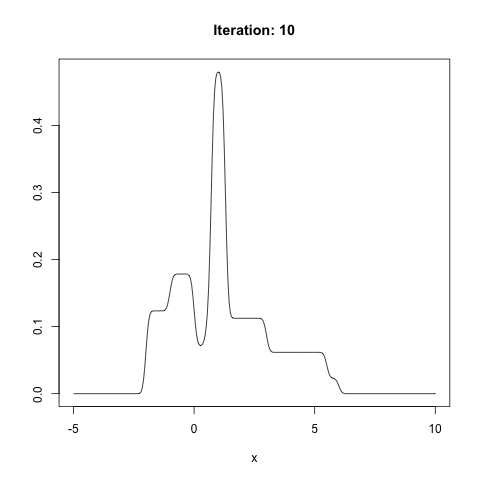


**a = 0.5**


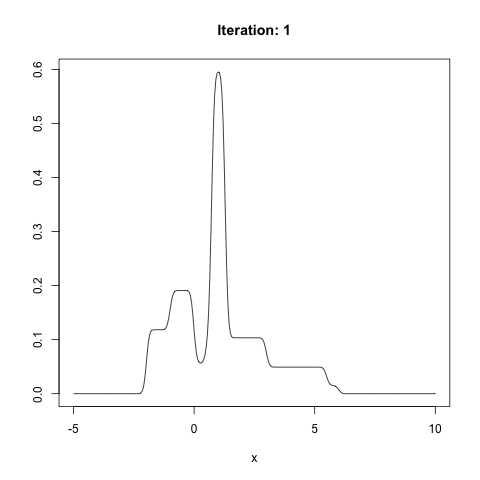

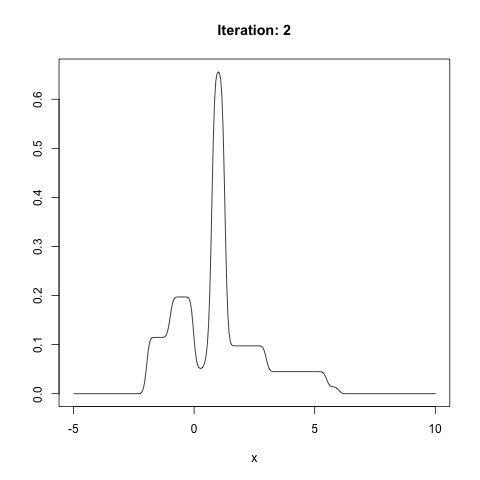

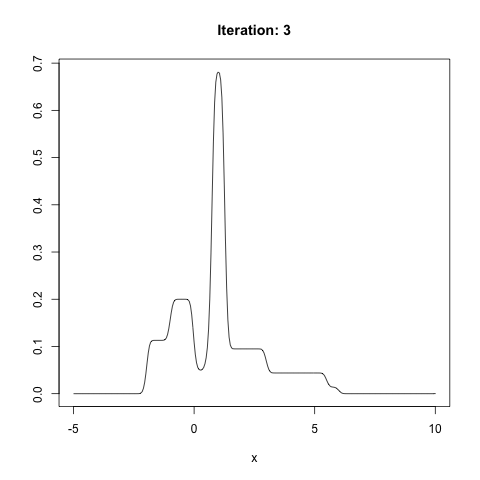

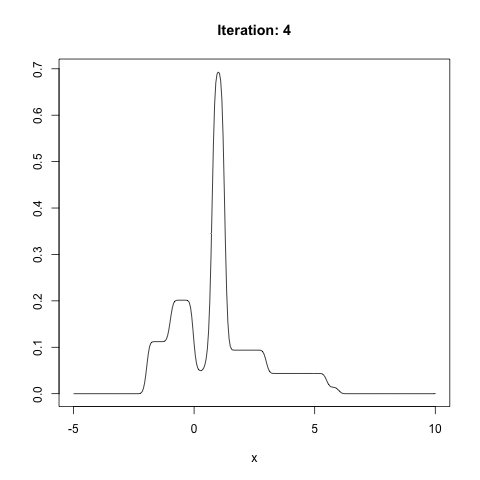
…
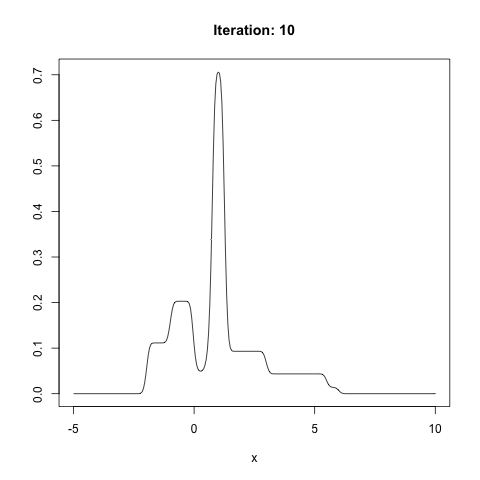


**a = 0.75**


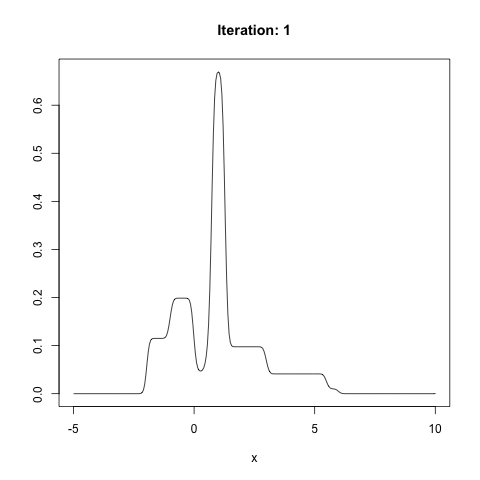

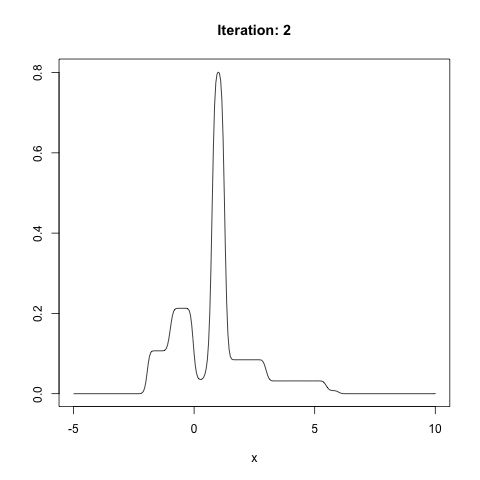


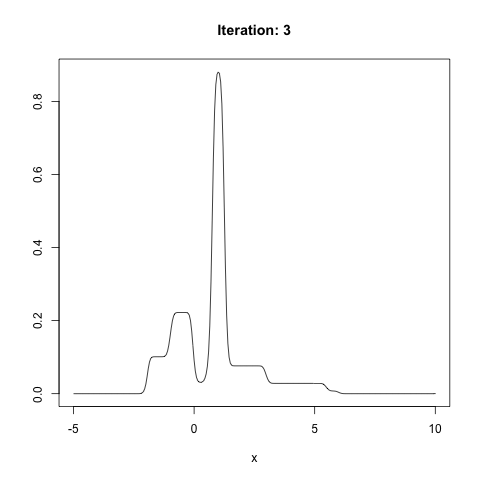

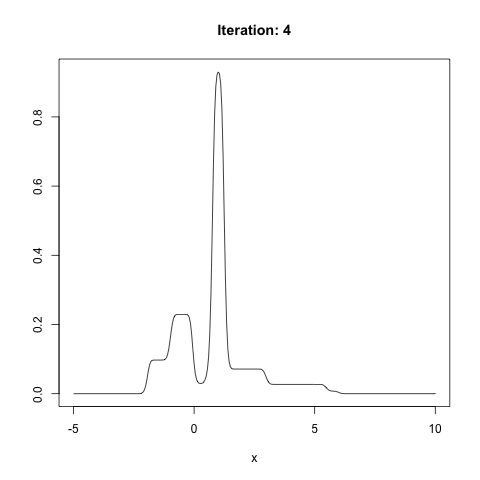

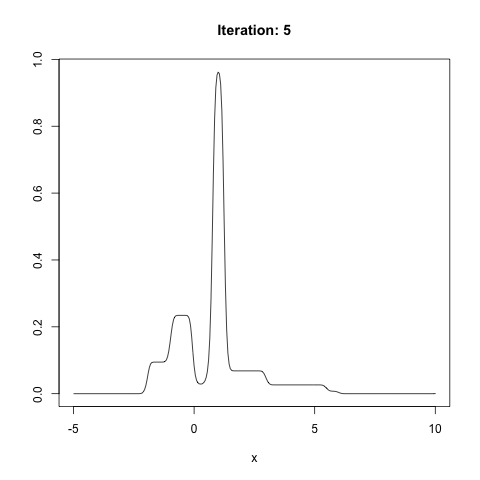

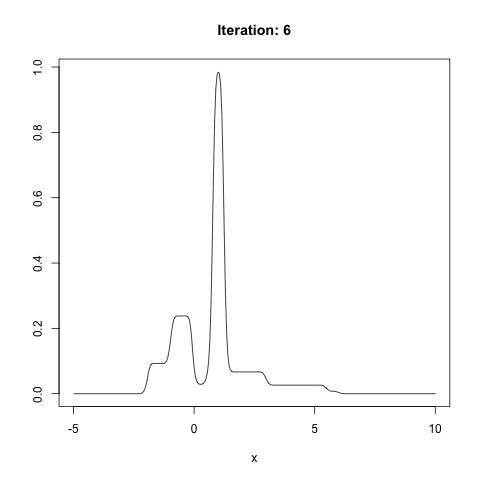

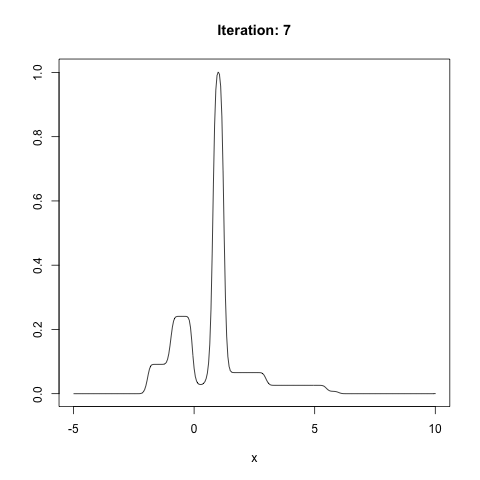
…
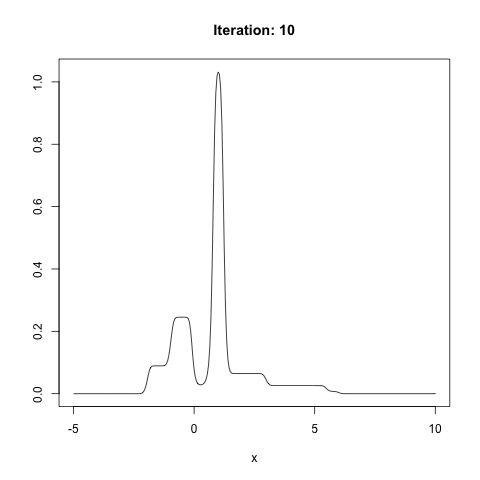

Supplement: Figure S1 — Numerical demonstration of convergence of equation (9) . A simple sample set 1-D of points was created with the following locations: −1, 1, 1, 2 and 3. A uniform contingency distribution was assumed for each point with radiuses of, respectively, 1, 2, 0.3, 4 and 2.5. The analytical method may only be used for the first estimate of f (i.e. f0(x)) as after that the contingent kernel estimates take on forms not tractable for analytical solutions. The initial contingent kernel density estimate is shown. Iterations were then carried out using different values of a. Values of 0.10, 0.50, and 0.75 are shown. Up to 10 iterations were carried out. Plots of iteration estimates of f are shown. Iterations are not shown if no significant visible changes were made between iterations. An ellipses sign marks these gaps. As is demonstrated, small values of a led to rapid convergence while values closer to 1 led to slower convergence. During iterations regions of high density increase in density while regions of low density generally decrease. (DOC) [file pone.0030549.s001.doc]
